# Micro computed tomography (Micro-CT) characterization of root and root canal morphology of mandibular first premolars: a systematic review and meta-analysis

**DOI:** 10.1186/s12903-023-03624-5

**Published:** 2024-01-02

**Authors:** Mohmed Isaqali Karobari, Rumesa Batul, Mohammad Khan, Santosh R. Patil, Syed Nahid Basheer, Nader Nabil Fouad Rezallah, Alexander Maniangat Luke, Tahir Yusuf Noorani

**Affiliations:** 1 Department of Restorative Dentistry & Endodontics, Faculty of Dentistry, University of Puthisastra, Phnom Penh, 12211 Cambodia; 2 Dental Research Unit, Centre for Global Health Research, Saveetha Institute of Medical and Technical Sciences, Chennai, 600077 Tamil Nadu India; 3 Conservative Dentistry Unit, School of Dental Sciences, Universiti Sains Malaysia, Health Campus, Kubang Kerian, Kota Bharu 16150, Kelantan, Malaysia; 4 New Age Health Science Research Center, Muradpur, Chattogram 4331 Bangladesh; 5 Department of Oral Medicine and Radiology, Chhattisgarh Dental College & Research Institute, Sundra, India; 6 Division of Operative Dentistry, Department of Restorative Dental Sciences, College of Dentistry, Jazan University, Jazan, Saudi Arabia; 7 Oral and Maxillofacial Radiology, City University Ajman, Ajman, UAE; 8 Department of Clinical Sciences, College of Dentistry, Ajman University, Ajman, UAE; 9 Center for Medical and Bio-Allied Health Sciences Research (CMBAHSR), Ajman University, Ajman, UAE

**Keywords:** Configuration, Dental anatomy, Dental pulp, Dental diagnostic imaging, Endodontics, Morphology, Micro-CT, Root, Root canal

## Abstract

**Introduction:**

Mandibular first premolars are familiar with their varied root canal morphology, causing difficulties and challenges for successful endodontic procedures. This systematic review and meta-analysis aim to study the characterization of root and canal morphology of the first mandibular premolar using micro-computed tomography.

**Methodology:**

The literature search was conducted using electronic web databases like PubMed, Scopus, ScienceDirect and Cochrane with the chosen MeSH key words and data was retrieved until May 2023. Further to perform the statistical analysis, R v 4.3.1 software with "meta", 'metafor" "metaviz" " ggplot2" package was used, and results were represented by odds ratios (OR) and the percentage of forest plots along a 95 per cent confidence interval (CI).

**Results:**

The total number of studies meeting the inclusion criteria was 13; these studies were conducted on mandibular first premolar using Micro-CT; the total sample size was 1817. To scan the sample, an X-ray micro-focus CT system (Siemens Inveon CT, Erlangen, Germany) was used in four studies and seven different machines were used in the respective studies. Mimics 10.01 software (Materialize, Leuven, Belgium) and NRecon v.1.6.9 software (Bruker, Kontich, Belgium) were commonly operated. The minimum and maximum voxel size ranges between 11.94 and 50 μm. Vertucci’s classification was frequently used (9), while one study applied Ahmed et al. and Vertucci’s classification.

**Conclusion:**

This systematic review provides essential information about the root and canal configurations, radicular grooves, accessory canals, and apical foramina through Micro-CT, aiming to improve the accuracy of endodontic treatment and help practitioners.

## Introduction

Knowledge of roots' external and internal anatomy and canals is critical to successful endodontic therapy [1]. Clinicians must be aware of teeth' normal and altered anatomy before any endodontic procedures to prevent possible accidents and avoid failure of root canal treatment [2].

The permanent mandibular first premolar is considered the most difficult tooth to treat due to the higher degree of root canal morphological variations and abnormalities, posing a greater challenge for the endodontic process [3]. These changes prevent the absolute obturation of total canals in the root canal system, complicating the treatment and affecting the possible outcome [4]. Variations can be due to differences in age [5], gender [1], ethnicity [6] and study design [5].

Root and canal morphology of the mandibular first premolar varied greatly, with a single root being predominant [7], but there are chances of two roots [8] and three roots [9]. The frequency of single canal and type I configuration is higher; however, multiple canals are found along with accessory canals and isthmus [10]. Other morphological variations include radicular grooves, C-shaped canals and Tome’s root [3].

Various methods are employed to evaluate the root and canal morphology of the mandibular first premolars. They are cross-sectioning and two-dimensional radiography [11], clearing technique using a dye [12], spiral computed tomography [13], cone beam computed tomography [9] and micro-computed tomography [10]. Micro-CT was developed by Elliott and Dover, an accurate research tool that provides precise details of root canal anatomy on higher resolution [14]. It is a non-invasive and non-destructive technique applied in endodontics for analyzing the internal anatomy of teeth, instrumentation and obturation of root canals, retreatment and evaluating the physical and biological properties of the materials [15]. It is the gold standard and reproducible method permitting the qualitative and quantitative characterization of teeth three dimensionally [10].

While our systematic review primarily focuses on the Micro-CT characterization of root and root canal morphology, it is crucial to acknowledge the broader landscape of diagnostic options available to dental professionals. In this context, it is worth noting that alternative methods have demonstrated their efficacy in providing valuable anatomical insights with distinct advantages.

One such approach that deserves special mention is the surgical-anatomical evaluation of mandibular premolars using CBCT, as exemplified in a recent study by Reda et al. Their research, conducted among the Italian population, showcases the potential of CBCT scans to offer comprehensive anatomical information while utilizing a reduced radiation dose. This approach aligns with the principles of minimizing radiogenic risks and enhances the precision of patient-specific treatment planning. It serves as a testament to the evolving landscape of diagnostic modalities, where patient well-being and treatment efficacy are paramount considerations [16].

Lack of accurate data and inconsistent information about root canal morphology can misguide the practitioner. Hence, systematic review is gaining importance as it can dispense and analyze information transparently. It facilitates the comparison among various quantitative and qualitative studies, thus presenting a high-quality outcome that can guide the clinician and research [17]. This systematic review aims to discuss the root canal characterization of the first mandibular premolars using micro-computed tomography.

## Methodology

### Protocol of the study

This review was in accordance with the PRISMA guidelines presented for systematic review and quantitative analysis. The present systematic review is registered with PROSPERO, and the registration number is CRD42023408084.

### Research questions

Research conducted on the evaluation of root and canal morphologies using microcomputed tomography was chosen as reported by the “PICOS” (PRISMA-P 2016) technique.

P (population): Extracted teeth.

I (intervention): Assessment by Micro-CT.

C (comparison): Characterization of the root canal morphology.

O (result): Quantitative analysis, root canal morphologies.

S (study design): Invitro studies.

### Search strategy

The search was carried out on electronic databases to identify the articles associated with evaluating root canal morphology of the first mandibular premolar through Micro-CT. Based on the selected key words, studies were searched without any limitation on the year until May 2023, and the acquired article from all databases is tabulated in Table 1. Studies were retrieved using MeSH keywords digitally on PubMed and Scopus. Later, the search was conducted on Cochrane and ScienceDirect databases to add more information. The literature search was imported using Endnote X8 software, and further duplicate studies were eliminated. Eligibility criteria were examined by screening the abstract, and the complete articles were obtained.

**Table 1:** Information of search strategies using MeSH keywords

Database	Search Strategies	Results
PubMed	(((((((((((((((((((((Tooth anatomy)) OR (Tooth Root)) OR (Tooth diagnosis)) OR (Tooth histology)) OR (Root canal morphology)) (Dental anatomy)) OR (Root canal system)) OR (Root canal configuration)) OR (Dental Pulp Cavity)) OR (Dental histology)) OR (Dental diagnostic imaging)) OR (Dental diagnosis)) AND (X-ray methods)) OR (X-ray Microtomography)) OR (micro computed tomography)) OR (Micro-CT)) OR (microcomputed tomography)) AND (mandibular first premolars))	30
Scopus	TITLE-ABS ("Tooth anatomy" OR "Tooth Root" OR "Tooth diagnosis" OR "Tooth histology" OR "Root canal morphology" OR "Root canal configuration" OR "Dental Pulp Cavity" OR "Tooth diagnostic imaging" OR "Dental diagnostic imaging") AND TITLE-ABS ( "X-ray methods" OR "X-ray Microtomography" OR "micro computed tomography" OR "Micro-CT" "microcomputed tomography") AND TITLE-ABS ( "mandibular first premolars")	11
Cochrane	"Tooth anatomy" OR "Tooth Root" OR "Tooth diagnosis" OR "Tooth histology" OR "Root canal morphology""Root canal configuration" OR "Dental Pulp Cavity" OR "Tooth diagnostic imaging" OR "Dental diagnostic imaging" AND "X-ray methods" OR "X-ray Microtomography" OR "micro computed tomography" OR "Micro-CT" OR "microcomputed tomography" AND "mandibular first premolars"	8
ScienceDirect	"Tooth Root" OR"Root canal configuration" OR "Tooth diagnostic imaging" OR "Tooth diagnosis" OR "Dental Pulp Cavity" AND "X-ray Microtomography" OR "microcomputed tomography" OR "Micro-CT" AND "mandibular first premolars"	10
Total		59

### Data extraction

Two researchers ( M.I.K and T.Y.N) conducted an electronic search on (19 May 2023) applying MeSH terms and keywords; also, to assemble the relevant information, Boolean operators like “OR” and “AND” were used with suitable filters. The keywords were "Tooth diagnosis", "Tooth Root", "Tooth diagnostic imaging", "Dental Pulp Cavity", "Root canal configuration", "X-ray Microtomography", "Micro-CT" "microcomputed tomography" and "mandibular first premolars". Further, the before-mentioned key words were combined using Boolean operators “OR” and “AND” with proper filters as described in Table 1.

### Eligibility criteria

**Fig. 1:**
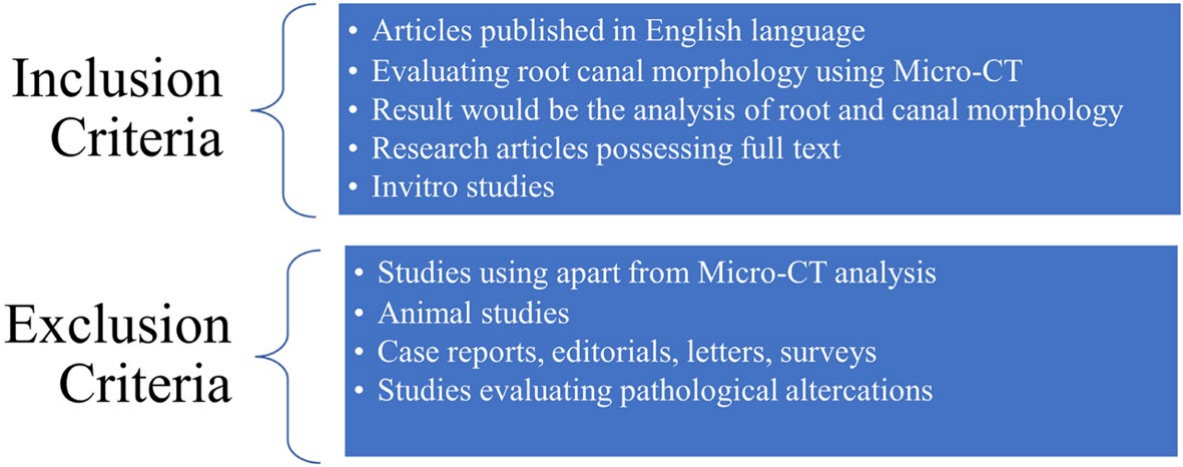
Inclusion and exclusion criteria of the studies

Invitro studies assessing the root and canal morphology of the first mandibular premolar through micro-CT was covered in the present systematic review. Two examiners used the PICOS approach to check the full text of papers and excluded the studies conducted on animals and studies issued in other than English language. The inclusion and exclusion criteria set by the examiners are illustrated in Fig. 1.

### Risk of bias and quality assessment of research articles

 Two authors (M.I.K and R.B) evaluated the total number of included studies based on the revised version of the earlier published risk of bias assessment tool [18]. This quality assessment was comprised of five objectives, and the result of each criterion was categorised as yes (adequate), unclear (not specified) and no (inadequate). The objectives of the risk of bias assessment are as follows:

i. Sample size calculation (yes, no, and unclear): sample size calculation is essential in any research to generalize the results and obtain a justifiable conclusion. Sample size calculator and G* POWER software are used to calculate it [19]. The sample size in the present study ranges from 50 [20] to 358 [21]. Sample size calculations were not mentioned in any of the included studies in this systematic review, which could be a week point. Further, the smaller sample size could negatively impact and mislead the results. However, the present review included micro-CT studies where various factors are associated with the studies like cost and maintenance of micro-CT machine, prolonged scanning time and smaller voxel size which could limit the inclusion of more samples [17]. Nevertheless, not conducting the sample size calculation or not reporting it, is the weak point.
ii. Reporting and quality of data (yes, no, and unclear): several factors affect the result and quality of data, like technique, scanning machine, voxel size and software used. When the parameters mentioned above are present, the study is adequate. The diagnostic machine and technique used are essential. Micro-CT machine is used in all the included studies, which has been an efficient tool in providing detailed and valuable data about the included samples [22].
iii. Result Description (yes, no, and unclear): The characteristics of the results are the evaluation of root canal configuration by different classifications, accessory canals, C-shaped canals, grooves, isthmus, foramen, and intercanal communication. All these features would form a valid result.
iv. Reliability of an observer (yes, no, and unclear): calibration is crucial in achieving definitive and validated results by minimizing error and decreasing the potential bias during data evaluation. Moreover, in micro-CT studies where data is qualitative, the evaluation process highly depends on the reliability of an observer [23].
v. Attrition bias (yes, no, and unclear) indicates sample loss. Here, sample loss does not refer to tooth loss but highlights the population that belongs to a definite region. A proper sampling technique was used in the study to cover the specific or mentioned population rather than generalizing it.

**Fig. 2:**
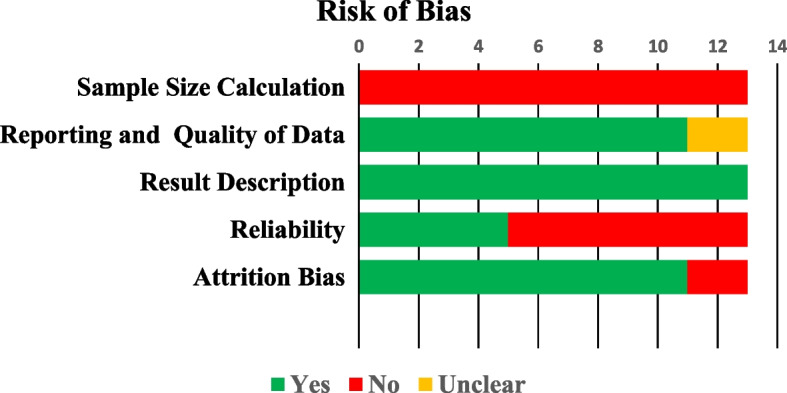
Risk of Bias of 13 included studies in the current review

A total of 13 articles were eligible to be part of this analysis. Data was extracted and are summarized in Fig. 2

**Fig. 3:**
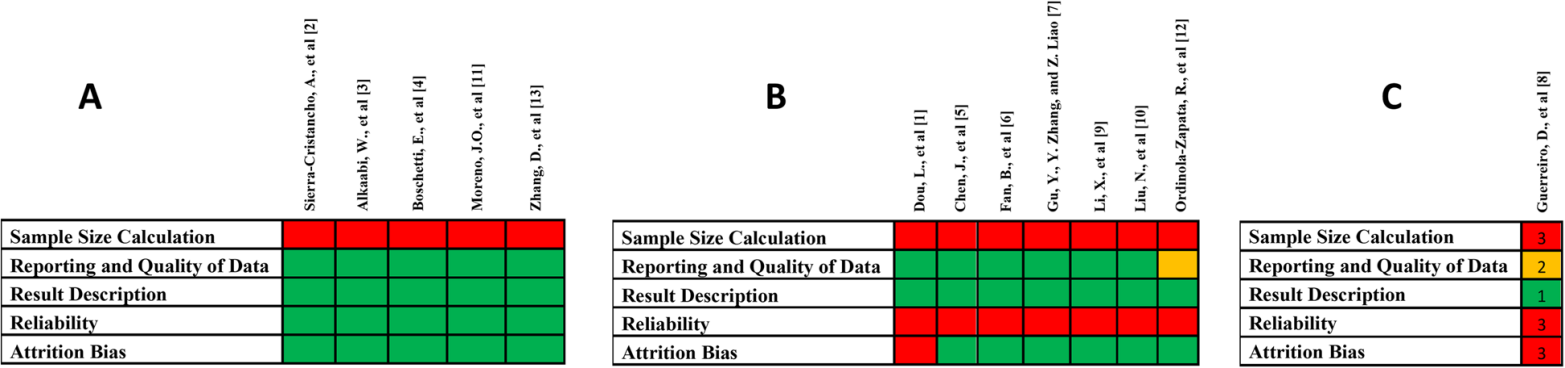
**A** Low risk of bias: studies that met at least four of the criteria; **B** Moderate risk of bias: studies that met between two and four of the criteria and **C** High risk of bias: studies that met less than two criteria. (Green-Yes; Red-No; Yellow-Unclear)

A. Low risk of bias (*i.e.,* studies meeting at least four of the assessment criteria): studies that met at least four of the quality criteria are classified under low risk of bias and are summarised in Fig. 3A
B. Moderate risk of bias (*i.e.,* studies meeting the criteria between two and four): Fig. 3B denotes the studies that met the assessment criteria between two and four.
C. High risk of bias (*i.e.,* studies meeting less than two assessment criteria): studies that fulfil less than two quality criteria were classified as high risk of bias and are shown in Fig. 3C

### Statistical analysis

The statistical analysis was conducted using R v 4.3.1 software with "meta", 'metafor", "metaviz", and " ggplot2" packages. The results were represented by odds ratios (OR) and the percentage of forest plots along a 95 per cent confidence interval (CI).

## Results

### Study selection outcomes

MeSH keywords were used to search the articles from different databases. Thirty research papers were yielded from PubMed, eleven using Scopus, eight from Cochrane, and ten from Science Direct. A total of 59 results were extracted from all the sources. Eight records were removed before screening as they were duplicates. Later, 51 articles were screened, out of which 28 research papers were excluded.

**Fig. 4:**
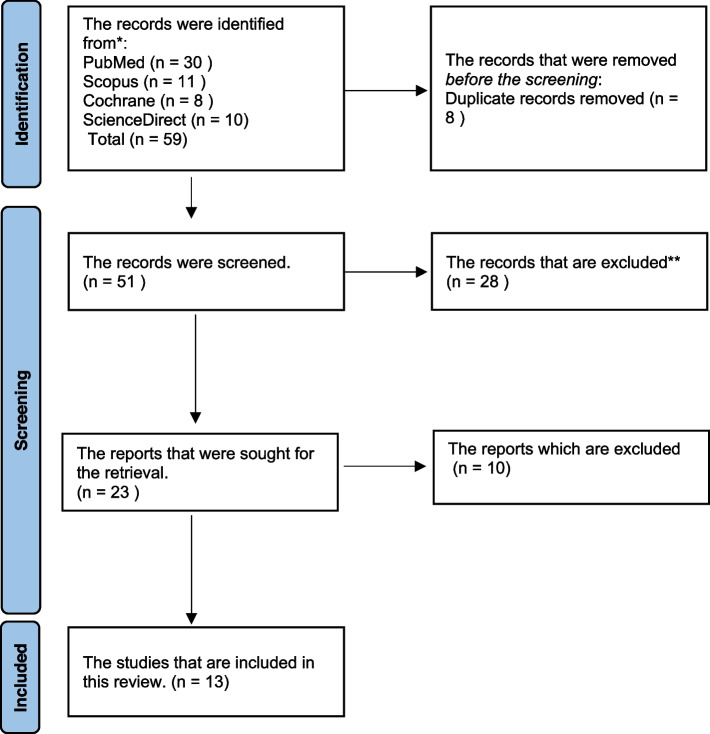
PRISMA flowchart

Further 23 reports were retrieved, and ten reports were then excluded. The final review included 13 studies after the eligibility assessment (Fig. 4). All the included studies were invitro studies. Figure 4 represents the selection criteria following PRISMA guidelines.

### Study features

The essential features of the included studies of the present systematic review are tabulated in Table 2. Studies that included different population lasted for varying periods between 2012 and 2021 were issued in different journals. Out of the 13 articles, population was mentioned in 12 studies. A higher number of studies were conducted on the Chinese (4) and Chinese-sub population (3), followed by Brazilian (2), Chilean (1), Emirati (1) and Columbian (1) populations.

**Table 2:** Included studies are as follows

No	Study reference	journal	Population	Year of publication
1	Dou, L., et al. [3]	Scientific reports	Chinese	2017
2	Sierra-Cristancho, A., et al. [10]	Scientific reports	Chilean	2021
3	Alkaabi, W., et al. [20]	Medical Principles and Practice	Emirati	2016
4	Boschetti, E., et al. [24]	Brazilian Dental Journal	Brazilian	2017
5	Chen, J., et al. [25]	Clinical Oral Investigations	Chinese	2014
6	Fan, B., et al. [21]	Journal of Endodontics	Chinese	2008
7	Gu, Y., Y. Zhang, and Z. Liao [26]	Archives of oral biology	Chinese	2013
8	Guerreiro, D., et al. [27]	Journal of Endodontics		2019
9	Li, X., et al. [28]	Journal of Endodontics	Southwestern Chinese	2012
10	Liu, N., et al. [29]	Clinical Oral Investigations	Southwestern Chinese	2013
11	Moreno, J.O., et al. [30]	Acta Odontológica Latinoamericana	Colombian	2021
12	Ordinola‐Zapata, R., et al. [31]	International Endodontic Journal	Brazilian subpopulation	2015
13	Zhang, D., et al. [32]	Clinical Oral Investigations	Southwestern Chinese	2017

The total number of mandibular first premolars included in the present systematic review is 1817 (Tables 3 and 4); sample size calculation was not mentioned in any of the involved studies. All the reported studies were in vitro studies. Ethical approval was taken by more than half of the mentioned studies (8 out of 13). All the articles mentioned the imaging details where a micro-CT scanning device was used. X-ray micro-focus CT system (Siemens Inveon CT, Erlangen, Germany) was used in four studies, SkyScan 1174v2; Bruker-microCT, Kontich, Belgium) in three studies (SkyScan 1278, Bruker, Kontich, Belgium) in one study, micro-CT scanner (micro-CT Inveon; Siemens Medical Solutions, Knoxville, TN) in one study, (Sky-Scan 1,172 X-ray micro-tomograph; SkyScan, Antwerp, Belgium) in one study, μCT-80; Scanco Medical AG, Brüttisellen, Switzerland) in one study, one article used micro-CT scanner (Inveon; Siemens Medical Solutions, Knoxville, TN) and μCT100; Scanco Medical, Bassersdorf, Switzerland was mentioned in one study.

**Table 3:** Characteristics of the involved studies are as follows

Author & year	Sample type	Ethical approval	Sample size	Estimation method	Technique	Diagnostic device	Voxel size	Software used	Classification used	Calibration
Dou, L., et al. [3]	Mandibular first premolar	Yes	178	Not mentioned	The extracted teeth sample were kept in sodium hypochlorite (5.25%) for one hour followed by removal of any remaining debris or calculus by scaling	(micro-CT Inveon; Siemens Medical Solutions, Knoxville, TN) micro-CT scanner	15 μm	Cobra software (Siemens Medical Solutions, Knoxville, TN)	Vertucci’s	-
Sierra-Cristancho, A., et al. [10]	Mandibular first premolar	Yes	186	Not mentioned	The extracted samples were placed in sodium hypochlorite (5%) for 30 min to clean, then kept in neutral buffered formalin (10%) followed by removal of debris by ultrasonic scaler. Samples were then stored in moisturizing solution until further analysis at room temperature	(SkyScan 1278, Bruker, Kontich, Belgium)	50 µm	NRecon v.1.6.9 software (Bruker, Kontich, Belgium), CTAn v.1.12 software (Bruker, Kontich, Belgium	Ahmed’s et al. and Vertucci’s classification	Yes
Alkaabi, W., et al. [20]	Mandibular first premolar	Yes	50	Not mentioned	Teeth were immersed in sodium hypochlorite (5.25%) to clean for 1 h followed by scaling. Samples were kept separately in the containers with 0.9% sodium chloride prior to analysis	Sky-Scan 1,172 X-ray micro-tomograph; SkyScan, Antwerp, Belgium)	11.94 μm	SkyScan software (CTan version 1.11.10.0	Vertucci’s classification	Yes
Boschetti, E., et al. [24]	Mandibular first premolar	Yes	70	Not mentioned	Extracted sample teeth were placed 0.1% thymol at 6℃, then the teeth were examined and analyzed	Bruker-microCT, Kontich, Belgium); SkyScan 1174v2	22.9 μm	CTAn v.1.16 software, NRecon v.1.6.9 software and CTVol v.2.3 sofware	Vertucci’s classification	Yes
Chen, J., et al. [25]	Mandibular first premolar	Yes	127	Not mentioned	Extracted teeth were cleaned to detach external tissues and scaling was done to remove the calculus followed by storing in neutral buffered formalin solution (10%) until analysis	X-ray micro-focus CT system (Siemens Inveon CT, Erlangen, Germany)	14.97 μm	Mimics 10.01 software (Materialize, Leuven, Belgium)	-	
Fan, B., et al. [21]	Mandibular first premolar	No	358	Not mentioned	All teeth were kept in 10% neutral buffered formalin, further debridement of any remnants was done before analysis followed by imaging	μCT-80; Scanco Medical AG, Brüttisellen, Switzerland)	37 μm	Image-Pro Discovery 5.0 (Media Cybernetics, Silver Spring, MD)	-	-
Gu, Y., Y. Zhang, and Z. Liao [26]	Mandibular first premolar	No	148	Not mentioned	Samples were stored in 10% formalin after removing calculus and any remnants of external tissues then followed by further micro-CT analysis	micro-CT scanner (Inveon; Siemens Medical Solutions, Knoxville, TN)	15 μm	Mimics 10.01 (Materialize, Leuven, Belgium)	Vertucci’s classification	-
Guerreiro, D., et al. [27]	Mandibular first premolar	Yes	154	Not mentioned	All the samples were placed in neutral buffered formalin (10%) after scaling was done to remove the calculus and any tissue remnants until analysis	μCT100; Scanco Medical, Bassersdorf, Switzerland	25 μm	_	Vertucci’s classification	-
Li, X., et al. [28]	Mandibular first premolar	No	115	Not mentioned	Samples were placed in neutral buffered formalin 910%) and sodium hypochlorite (5%) was used to clean for 24 h. Later calculus or any external tissues were removed	X-ray microfocus CT scanner (Siemens Inveon CT, Erlangen, Germany)	14.97 μm	Mimics 10.01 software (Materialise, Leuven, Belgium)	Vertucci’s classification	-
Liu, N., et al. [29]	Mandibular first premolar	No	115	Not mentioned	Extracted teeth were separately kept in neutral buffered formalin (10%) and sodium hypochlorite (5%) was used to clean the samples for 24 h. Thorough debridement of calculus and periodontal tissues was done until micro-CT assessment	X-ray microfocus CT scanner (Siemens Inveon CT, Erlangen, Germany)	14.97 μm	Mimics 10.01 software (Materialise, Leuven, Belgium)	Vertucci’s classification	-
Moreno, J.O., et al. [30]	Mandibular first premolar	Yes	50	Not mentioned	-	SkyScan 1174v2 micro-CT device (Bruker-microCT, Kontich, Belgium)	17 μm	NRecon v.1.6.9 software (Bruker-micro-CT), CTAn V.1.13 software (Bruker-micro-CT) and CTVol v.2.2.1 (BrukermicroCT)	Vertucci’s classification	Yes
Ordinola‐Zapata, R., et al. [31]	Mandibular first premolar	-	123	Not mentioned	-	(SkyScan 1174; Bruker-microCT, Kontich, Belgium)	19.6 μm	NRecon 1.6.3 software (Bruker micro-CT), CTAn v.1.12 software (Bruker microCT) and Data viewer software (Bruker-micro-CT)	-	-
Zhang, D., et al. [32]	Mandibular first premolar	Yes	143	Not mentioned	Samples were disinfected using 5.25% sodium hypochlorite to detach the calculus and debride any external tissues followed by storage of extracted teeth in 10% neutral buffered formalin until further assessment	X-ray microfocus CT scanner (Siemens Inveon CT, Erlangen, Germany)	14.97 μm	Mimics10.01 software (Materialise, Leuven, Belgium)	Vertucci’s classification	Yes

**Table 4:** Outcome of the studies

Reference	Results	Grooves	Canal configuration			Accessory canals	C shapes anatomy	Isthmus	Foramen	Intercanal communication	Conclusion
Dou, L., et al. [3]	All the mandibular first premolars included in the study were single rooted except for two (0.6%) where two roots were found Canals: 1 canal: 64.04% 2 canals: 34.27% 3 canals: 1.69% Other findings were radicular grooves and C shaped canals. Grade 3 and 4 radicular grooves were termed as Tome’s anomalous roots	Radicular grooves were noticed among 44.34% of total samples **Distribution according to tooth surface** Mesial: 41.57%, distal:2.81%, lingual:2.81%, buccal:1.12%, One proximal &two lingual: 1.67% Two proximal and lingual: 1.12% **Scoring according to ASUDAS** Grade 0: 56.74%, grade 1: 16.85%, grade 2: 12.36%, grade 3: 10.11%, grade 4: 3.37% and grade 5: 0.56%	Vertucci’s classification: Type I: 64.04% Type II: 1.12% Type III: 10.67% Type IV: 0.56% Type V: 21.91% Type VIII: 1.12% Type (1–3-1): 0.56%			Lateral canals were found in 39.89% of teeth samples **Location** Coronal: 5.95% Middle: 46.43% Apical: 47.62% **Apical delta** was seen in 10.11% of teeth	C shaped canals were noted in 12.36% of teeth	_	Apical foramen: Single: 76.4% Double: 23.6% Central:53.37% Lateral:46.63%	10.67% of teeth had intercanal communication **Location** Coronal: 10% Middle: 60% Apical: 30%	Multiple canals and varied root canal morphology was observed which specified the need of careful examination for successful endodontic procedure
Sierra-Cristancho, A., et al. [10]	Results stated that 99.46% of teeth had one root and two roots were found in only one tooth. Radicular grooves, accessory canals, C shaped canals and isthmus were located. Tome’s anomalous root was found only in multiple root canals teeth whereas teeth with one root canal had no evidence of them	Radicular grooves as stated by **ASUDAS scoring:** Grade 0: 60.75% Grade 1: 13.98% Grade 2: 12.36% Grade 3: 10.22% Grade 4: 2.15% Grade 5: 0.54% Non tomes root: 74.69%	Type	Vertucci’s classification	Ahmed’s et al. classification	Accessory canals were noticed in 62.90% of total teeth sample **Location of accessory canals** Coronal: 0% Middle: 6.99% Apical: 43.55% Middle and apical: 12.37% **Apical delta** was found in 26.88% of first mandibular premolar	29.57% of samples possessed C shaped canals Coronal: 0% Middle: 6.99% Apical: 3.23% Combining middle and apical: 19.35%	Percentage of teeth samples according to the location of isthmus is as follows: Coronal: 05 Middle: 19.35% Apical: 9.68% Middle & apical: 2.69%	One: 36.56% Two: 27.42% Three: 15.59% Four: 20.43% **Location of apical foramen** Central: 37.63% Lateral: 62.37%	_	Variations in roots and canals were noticed. Radicular groves were found among the teeth with complex root anatomy and multiple canals. Tome’s anomalous roots were present in the roots with multiple canals
			Type I Type II Type III Type IV Type V Type VI Type VII Type VIII Type IX Non classified:	65.05% 0% 5.38% 0% 24.19% 0% 2.15% 0% 2.69% 0.54%	65.05% 0% 5.38% 0% 24.19% 0% 2.15% 0% 0.54% Double rooted: 0.54%						
Alkaabi, W., et al. [20]	Mandibular first premolar had varied root canal configuration, two additional types of canal configuration type 1–2-3 and type 1–3 which were not classified by Vertucci’s criteria were found. Further lateral canals, C shaped canals, apical delta, apical foramen, inter canal communication and apical loops were assessed	Out of the scanned samples, 20% of them had deep grooves on mesiolingual side and distal side had shallow depressions. Further a groove was found on either mesial or distal surface in 24% of specimen, later superficial depression was noticed among 24% of teeth on each of two -proximal surfaces	Vertucci’s classification: Type I: 62% Type III: 2% Type IV: 4% Type V: 20% Type VI: 4% Type VII: 2% Extra canal type: 6%			Lateral canals were found in 44% of included teeth with increased incidence in the apical region of root	C shaped canal configuration appeared in 28% of sample teeth	_	Apical foramina were 70 amongst examined teeth **Numbe**r One: 76.6% Two: 16.6% Three: 6% **Position** Centra: 37.2% Lateral: 62.8% **Apical delta** was present in 60% of examined specimen	Intercanal canal communication was recognized in 12% of sample teeth	It was concluded from the study that, involved population had complex root canal morphology with higher incidence of multiple root canals and their configuration
Boschetti, E., et al. [24]	Root canal morphology and radicular grooves were evaluated Canal orifices ranged from 1 to 4 and higher frequency of two canal orifices were seen in apical third of teeth (63.7%). Mean volume (10.78 m ${m}^{3}$), surface area (58.51${mm}^{2}$) and structure model index was determined. Mean length of radicular groove and root were 8.5 mm and 13.43 mm individually. Depth of radicular groove was different and deeper across the root length. At the half level of radicular groove length, the mean dentinal thickness in mesial or distal surface of the root was ranging between 1.0 to 1.31 mm	**Location of radicular grooves:** Mesial: 95.70% Distal: 2.85% Vestibular: 1.45% **ASUDAS scoring:** When canals are not divided the scoring is as follows Grade 2: 11.42% Grade 3: 1.43% Grade 4: - When canals are divided Grade 2: 67.15% Grade 3: 17.15% Grade 4: 2.85%	Vertucci’s classification Type I: 12.85% Type III: 11.43% Type V: 58.57% Type VII: 10%			One accessory canal was 20 in number that emerged from middle third and 18 from apical third Two accessory canals were only two in number emerging from middle third and 14 from apical third. **Apical delta** was seen in 4.35% of total examined teeth	C shaped root canal configuration was noticed in 18.57% of assessed teeth	_	_	_	The study concluded wide range of variations of radicular grooves. Anatomical complexities like C-shaped canals and division of the main root canal were noticed
Chen, J., et al. [25]	Radicular grooves were assessed which stated that 40.9% of included sample teeth had RG most of which were present on mesial side of root (69.5%) and their incidence was higher in multiple root canals than single canal. Depth and mean length of radicular grooves were apparently deeper and longer in type V and other canal configurations than type I	Distribution according to type of canals: Type I: 17.4% Type III, V and other complex type: 90.2% Most grooves were present on the mesial side (69.5%) of root. 84% of them began at the coronal third and similar percentage of them terminated at the apical third, further extension to the apex was shown by 40.8% of the grooves. Mean length of grooves: Type I: 6.06 ± 2.12 mm Type V: 7.70 ± 2.16 mm Other types: 6.91 ± 2.67 mm	_			_	_	_	_	_	The morphology of the root canal was influenced by the radicular groove’s anatomy. Hence it was stated that multiple canals and complex root morphology had higher prevalence of radicular grooves than simple and single root canals
Fan, B., et al. [21]	Results stated the morphology of radicular grooves and anatomy of C shaped root canals. The majority of grooved were present on the mesiolingual side of the root and C shaped canals varied greatly in shape. Frequency of two canals was higher at apical (80%) than middle (20%)	Distribution of the groves according to surfaces: 73 grooves were present on **the mesial lingual** side, out of which 66 were single and 7 were double, 10 grooves were found on **distal lingual,** and they were single, 3 single grooves were present on **lingual** side and 7 double grooves were found on **buccal** surface Mean distance of the groove from coronal top plane to CEJ was 3.46 mm. Similarly, mean distance from apical bottom plane to the apex was 1.38 mm. extension of the grooves to the apex was 43% and 8.77 mm was the mean length of grooves. The ratio of length of the grooves to the length of the root was 65%. Mean distance and mean depth of the grooves were 5.41 and 1.44 mm respectively	_			_	C shaped canals were classified as follows: C1: continuous c shape C 2: semicolon shape due to discontinuous outline C3: two different canals with round, oval or flat shape C4: one canal with round, oval or flat shapes C4a: long diameter of canal is equal to short diameter of round canal C4b: long diameter of canal was two times smaller than short diameter of oval canal C4c: long diameter of canal was two times bigger than short diameter of flat canal C5: three or more different canals C6: no intact canal C4b and C4c were usually single canal present at coronal third, however C1, C2 and C3 were higher at middle third. Gradually C1 reduced apically but C2 and C3 were found	_	_	_	Study stated that, the radicular grooves may present relative morphological changes in the presence of C-shaped root
Gu, Y., Y. Zhang, and Z. Liao [26]	Micro-CT examination of sample was performed to assess the root canal morphology, C shaped canals radicular grooves and Tome’s root. Out of the total sample 81.1% were non-Tome’s root, 18.9% of sample had Tome’s root 0.4% were double rooted premolars. Further accessory canals and canal communication was also detected	155 radicular grooves were found among the examined teeth, and they were mostly observed on mesiolingual side. Shallow grooves were 37.5% and deep grooves were 18.5% **Mean depth and angle of mesial radicular groove:** ASU (1): 0.18 mm, 28.8∘ ASU (2): 0.36 mm, 47.5∘ ASU (3): 1.24 mm, 101.∘7 ASU (4): 0.95 mm, 87.0∘ **Mean depth and angle of distal radicular groove:** ASU (1): 0.27 mm, 29.3∘ ASU (2): 0.21 mm, 25.4∘ ASU (3): 0.13 mm, 18.1∘ ASU (4): 0.55 mm, 59.2∘ The mean length of root was 12.98 ± 1.36 mm and the mean angle and depth of concavities were 21.0∘ and 0.20 mm ASUDAS scoring (3–5) represents the Tome’s root, seen in 18.9% of teeth	Higher number of Type I canal configuration had ASUDAS scoring 0,1 and 2, non-Tome’s root had 78.2% of Type I canal configuration while 8.5% of Type I had Tome’s root Further complicated canal configuration ranges between 15.5% (ASU-0) to 100% (ASU-4 & 5)			Accessory canals were found in 48% of teeth and 19.6% of teeth had lateral canals, similarly apical delta was found in 37.8% of teeth. 10 accessory canals had communication between radicular groove and the main canal	19.6% of examined samples had C shaped canals and were categorized into C1 (44), C2 (42) and C3 (21). The majority of C1 and C2 canals were 7–9 mm distance away from the CEJ	_	_	_	Severity of radicular grooves determine the complexity of root and canal morphology which was scored by ASUDAS. Hence success of an endodontic treatment relies on understanding the internal anatomy of root and radicular grooves
Guerreiro, D., et al. [27]	Radicular groove accessory canals (RGAC) were assessed, which shows their presence in 49.9% of teeth and were predominantly present in the middle third. They were majorly found in ASUDAS group 3 and 4 and had different vertucci’s configuration of the root canal	Relationship between presence of RGAC and ASUDAS scoring: Grade 1: 3.2% Grade 2: 11.1% Grade 3: 68.3% Grade 4: 17.5%	Relationship between presence of RGACs and canal morphology (Vertucci’s classification): Type I: 0% Type III: 1.6% Type V: 46% Type VII: 6.3% Non classified (others): 46%			49.9% of radicular groove accessory canals were found upon examination, out of which 53.9% originated from semilunar buccal canal, 46.1% from pulp chamber extension to radicular groove	_	_	Out of the assessed teeth that showed the presence of RGACs, single foramen was seen in 74.6% of teeth at the groove, two foramina in 11.2% and three or more foramina in 14.3% of specimen. Their incidence was higher in the mid third of the root The average diameter of RGAC foramen was 0.088 ± 0.048, similarly average distance was 8.83 ± 2.53 from CEJP to FP at the groove	_	The present study derived an association between radicular groove accessory canals and internal and external teeth anatomy, and it was also concluded that accessory canals, radicular groove are often present in the mandibular first premolars with radicular grooves
Li, X., et al. [28]	The lingual canal of mandibular first premolar was examined, 69% of them were present in the middle third and the remaining were found in apical third of the tooth. However, lingual view showed 73% of canals in the middle third and remaining in the coronal third In proximal view, mean angle α and β were 33.54∘ and 26.66∘ and angles were extremely curved while in the lingual view, they were less curved with mean angle α 8.31∘ and β 11.31∘ respectively	_	_			_	_	_	_	_	The study provided the details of lingual canal, however the information obtained was influenced by the view used
Liu, N., et al. [29]	Micro-CT assessment of first mandibular premolar was performed to analyze the root and canal morphology, accessory canals, intercanal communication, isthmus and apical foramina Teeth with mesial invaginations (27.8%) had multiple canals and majority of them were in the middle third of the root	_	Vertcci’s classification: Type I: 65.2% Type III: 2.6% Type V: 22.6% Type VII: 0.9% 1–3-2: 2.6% 1–3: 5.2% 1–2-3: 0.9% Mesial invagination was present in 27.8% (32) of teeth, they were classified according to vertucci’s criteria as: Type I: 2 Type III: 2 Type V: 21 Type VII: 1 1–2-3: 1 1–3: 2 1–3-2: 3			Teeth with accessory canals were 35.7% Number of accessory canals: 1–87.8% 2–9.8 3–2.4% Location of accessory canals: Coronal: 0 Middle: 3 Apical: 38 Apical delta was seen in 6.1% of teeth	_	Only 2 isthmuses were observed, one in the middle and the other in the apical third of teeth Loops were 8 in number, three in the middle and five in the apical third of specimen	Out of the assessed teeth that showed the presence of RGACs, single foramen was seen in 74.6% of teeth at the groove, two foramina in 11.2% and three or more foramina in 14.3% of specimen. Their incidence was higher in the mid third of the root The average diameter of RGAC foramen was 0.088 ± 0.048, similarly average distance was 8.83 ± 2.53 from CEJP to FP at the groove	Intercanal canal communication was recognized in 12% of sample teeth	Micro-CT scanning of the mandibular first premolar among the included population revealed the complex morphology of the root with multiple canals and varied canal configurations
Moreno, J.O., et al. [30]	Results analyzed canal configuration according to vertucci’s criteria and C shaped canals (1.8%) Mean values of parameters at 1,2 and 3 mm from apical foramen are as follows: Perimeter in mm: 1.07 ± 0.57, 1.27 ± 0.78 and 1.57 ± 0.84 Circulatory in mm: 0.59 ± 0.19, 0.57 ± 0.20 and 0.56 ± 0.22 Major diameter in mm: 0.41 ± 0.23, 0.48 ± 0.33 and 0.60 ± 0.37 Minor diameter in mm: 0.24 ± 0.10, 0.26 ± 0.11 and 0.21 ± 0.13 The overall area and volume are 61.27 ± 16.47 mm2 and 12.47 ± 4.95 accordingly	_	Vertucci’s classification: Type : 40% Type III: 4% Type V: 24% Type VII: 4% Additional types: 1–3: 3 1–4: 1 1–2-3: 6 1–3-2: 1 1–2-1–2: 1 1–2-1–2-1–3: 1 1–2-4–3-4–3: 1			_	_	_	_	_	The study concluded wide variations of the involved teeth among Colombian population emphasizing the need for appropriate anatomical knowledge of the teeth to establish more effective endodontic treatment
Ordinola‐Zapata, R., et al. [31]	All the mandibular first premolars had one root except for three teeth where two roots were found C -shaped roots were present in 67.47% mandibular first premolars. Mean distances were in the range of 5.36–5.65 mm from furcation to CEJ	_	Vertucci’s classification: Type I: 13% Type III: 8% Type V: 37% Type VII: 2% 1–2-3: 8% 1–2-3–2: 7% 1–2-3–2-3: 2% 1–2-1–2-4: 1%			Apical delta was present in 43% of specimen and furcal canals were noticed in 33%	67.47% of specimen revealed C-shaped roots and C-shaped canal was found only in buccal canal.C1 and C2 type of canals were frequent in the middle and apical middle levels whereas cervical third commonly had C4c cross section	_	_	_	The study concluded that 67% of the specimen had C-shaped canals with radicular grooves. Vertucci’s type I and V were more common. C-shaped cross section was frequently noticed in the middle third
Zhang, D., et al. [32]	136 mandibular first premolars were examined, and root canal morphology was evaluated by vertucci’s classification system	_	Type I: 74.13% Type III: 3.50% Type V: 13.99% Type V11: 0.70% Type IX: 7.69% Additional types 1–3: 7 1–2-3: 2 1–3-2: 2			_	_	_	_	_	Micro-CT provided more detailed anatomical information of the included mandibular first premolar teeth along with root and canal variations

Voxel size was specified in 12 articles ranging between 11.94–50 μm. Out of the 13 studies, 12 articles mentioned the particulars of software used where Mimics 10.01 software (Materialize, Leuven, Belgium) was operated in five studies, NRecon v.1.6.9 software (Bruker, Kontich, Belgium) in three, Cobra software (Siemens Medical Solutions, Knoxville, TN) in one study, SkyScan software (CT a version 1.11.10.0 in one study, Image-Pro Discovery 5.0 (Media Cybernetics, Silver Spring, MD) in one study and NRecon 1.6.3 software (Bruker micro-CT) was used in one study. 9 out of 13 studies classified the samples using Vertucci’s classification; one used both Vertucci’s and Ahmed et al. classifications, while three did not mention any details. Calibration was performed only in one study using the kappa test.

### Radicular grooves

**Fig. 5:**
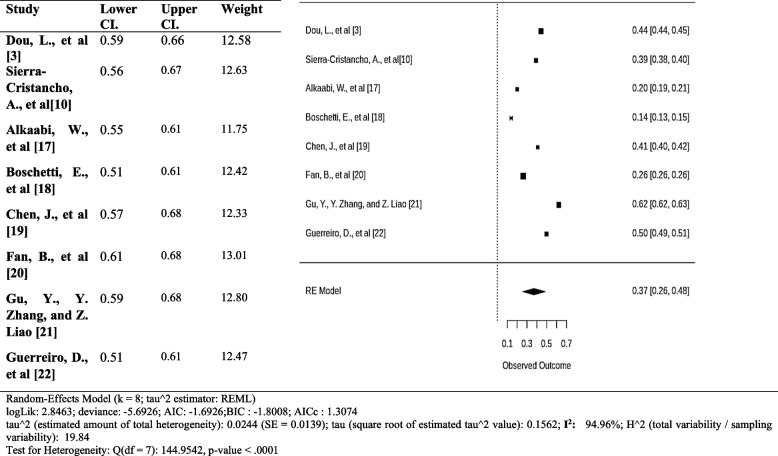
Forest plot for radicular grooves in mandibular first premolars

The forest plot shows the estimated prevalence of radicular grooves in each study and the 95% confidence intervals (CIs) for each estimate. The pooled frequency of radicular grooves is 11.4% (95% CI: 9.5%, 13.5%). This means that, based on the results of this meta-analysis, approximately 11.4% of premolars have radicular grooves. The forest plot also shows the results of a test of heterogeneity. The heterogeneity test was significant, meaning there is significant variation in the prevalence estimates across the studies. This implies that the incidence of radicular grooves in premolars is likely to be similar across all studies. Overall, it can be suggested from the results of the present meta-analysis that the prevalence of radicular grooves in 1^st^ premolars is relatively high (Fig. 5).

### Root canal variations

#### Occurrence of Type I Vertucci canal configuration

**Fig. 6:**
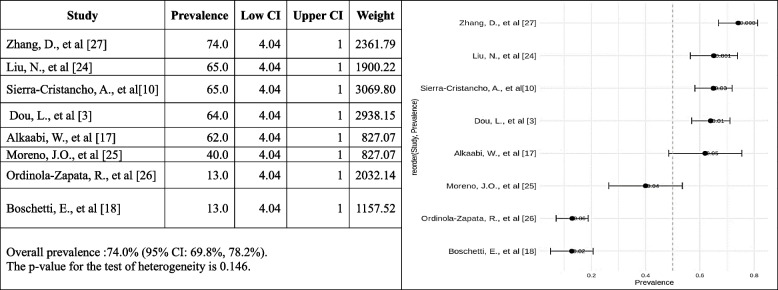
Forest plot of prevalence of Vertucci type I canal configuration

The forest plot shows the estimated occurrence of Vertucci type I in each study and the 95% confidence intervals (CIs) for each estimate. The pooled prevalence of Vertucci type I is also shown, along with the 95% CI for the pooled estimate. The pooled occurrence of type I Vertucci canal configuration is 74.0% (95% CI: 69.8%, 78.2%). This means that, based on the results of this meta-analysis, 74.0% of people have Vertucci type I root canal configuration. The heterogeneity test was not significant, meaning there is no significant variation in the prevalence estimates across the studies. This suggests that the occurrence of Vertucci type I root canal configuration is likely similar across all studies. The studies in the following meta-analysis were performed in different countries, so there may be some variation in the prevalence of Vertucci type I canal configuration across different populations. However, the lack of significant heterogeneity suggests this variation is likely slight. Overall, it can be recommended from the results of this meta-analysis that the prevalence of Vertucci type I canal configuration is high. This is consistent with previous studies investigating the incidence of Vertucci type I root canal configuration (Fig. 6).

#### Occurrence of Vertucci type III root canal configuration

**Fig. 7:**
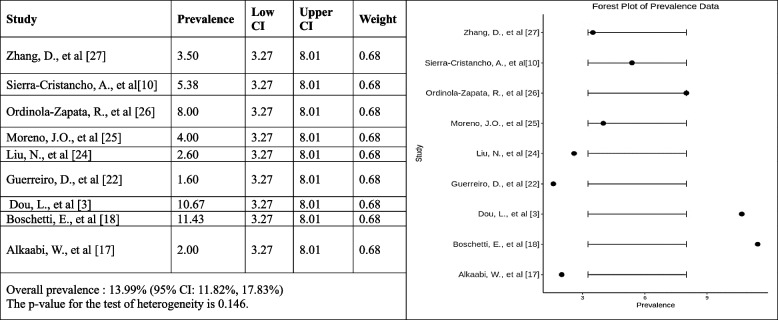
Forest plot of prevalence of Vertucci type III canal configuration [3, 10, 18, 20, 22, 24, 33–35]

The forest plot shows the estimated prevalence of Vertucci type III in each study and the 95% confidence intervals (CIs) for each estimate. The pooled incidence of Vertucci type III is also shown, along with the 95% CI for the pooled estimate. The pooled prevalence of Vertucci type III canal configuration is 13.99% (95% CI: 11.82%, 17.83%). This means that, based on the results of this meta-analysis, 13.99% of people have Vertucci type III canal configuration. The heterogeneity test was not significant, meaning there is no significant variation in the prevalence estimates across the studies. This suggests that the prevalence of Vertucci type III canal configuration is likely similar across all studies. Overall, the results suggest that the prevalence of Vertucci type III canal configuration is relatively low. This is consistent with previous studies investigating the prevalence of Vertucci type III canal configuration (Fig. 7).

#### Occurrence of Vertucci type V root canal configuration

**Fig. 8:**
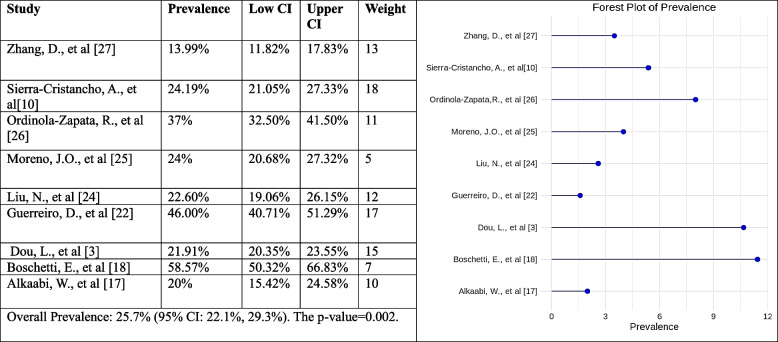
Forest plot of prevalence of Vertucci type V canal configuration [3, 10, 18, 20, 22, 24, 33–35]

The forest plot shows the estimated incidence of type V Vertucci canal configuration in each study and the 95% confidence intervals (CIs) for each estimate. The pooled occurrence of Vertucci type V is also shown, along with the 95% CI for the pooled estimate. The pooled prevalence of Vertucci type V is 25.7% (95% CI: 22.1%, 29.3%). This means that, based on the results of this meta-analysis, 25.7% of people have 1st premolar configuration of Vertucci type V. The heterogeneity test was significant (*p* < 0.05), meaning there is significant variation in the prevalence estimates across the studies. Overall, the result of the present meta-analysis implies that the prevalence of 1st premolar configuration of Vertucci type V is 25.7%, and there is a statistically significant difference between the studies (Fig. 8).

#### Prevalence of Vertucci type VII canal configuration

**Fig. 9:**
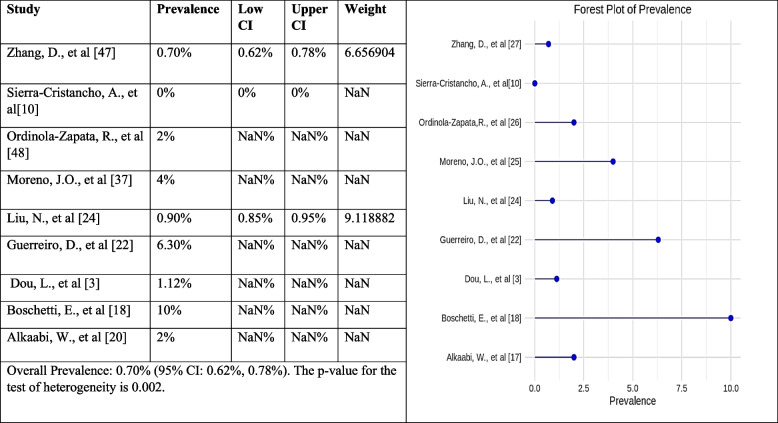
Forest plot of prevalence of Vertucci type VII canal configuration [3, 10, 18, 20, 22, 24, 33–35]

The forest plot shows the estimated prevalence of Vertucci type VII in each study and the 95% confidence intervals (CIs) for each estimate. The pooled frequency of Vertucci type VII is 0.70% (95% CI: 0.62%, 0.78%). This means that, based on the results of this meta-analysis, 0.70% of people have Vertucci type VII canal configuration. The forest plot also shows the results of a test of heterogeneity. The heterogeneity test was significant, meaning there is significant variation in the prevalence estimates across the studies. Several factors could be responsible for these variations, like variations in the studied populations, the methods applied to assess canal configuration, and the quality of the studies. Overall, it was suggested from the results of the present meta-analysis that there is notable variation in the prevalence of Vertucci type VII canal configuration (Fig. 9).

#### Prevalence differences between Vertucci Types and canal variations

**Fig. 10:**
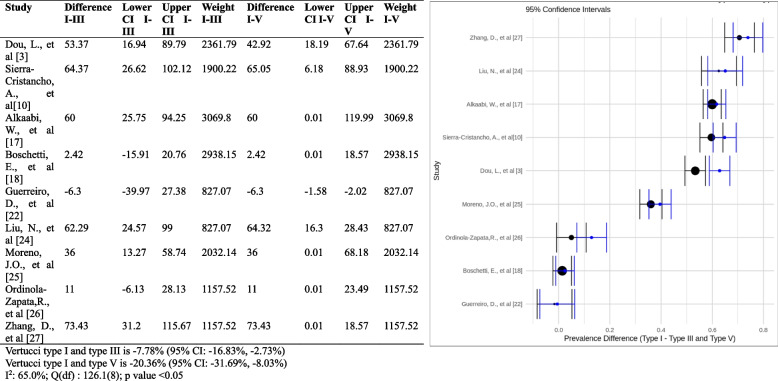
Forest plot of prevalence differences between Vertucci Type I vs Type III and Type V [3, 10, 17, 18, 22, 24–27]

The forest plot shows the estimated difference in prevalence between type I, type III, and type V Vertucci canal configuration in each study, as well as the 95% confidence intervals (CIs) for each estimate. The pooled difference in prevalence between type I and type III is -7.78% (95% CI: -16.83%, -2.73%). This means that the results of the present meta-analysis suggest a statistically notable difference in the prevalence of Vertucci type I and type III canal configuration, with type I being more prevalent. The pooled difference in prevalence between Vertucci type I and type V is -20.36% (95% CI: -31.69%, -8.03%). This means that, based on the outcome, there is a statistically significant difference in the prevalence of Vertucci type I and type V root canal configuration, with Vertucci type I being more prevalent. The forest plot also shows the results of a test of heterogeneity. The heterogeneity test was significant, meaning there is significant variation in the prevalence difference estimates across the studies. This suggests that the prevalence difference between Vertucci type I, type III and type V root canal configuration is likely to be similar across all studies. Overall, it can be suggested from this meta-analysis that there is a notable statistical difference in the prevalence of Vertucci type I, type III and type V canal configuration, with type I being more prevalent (Fig. 10).

**Fig. 11:**
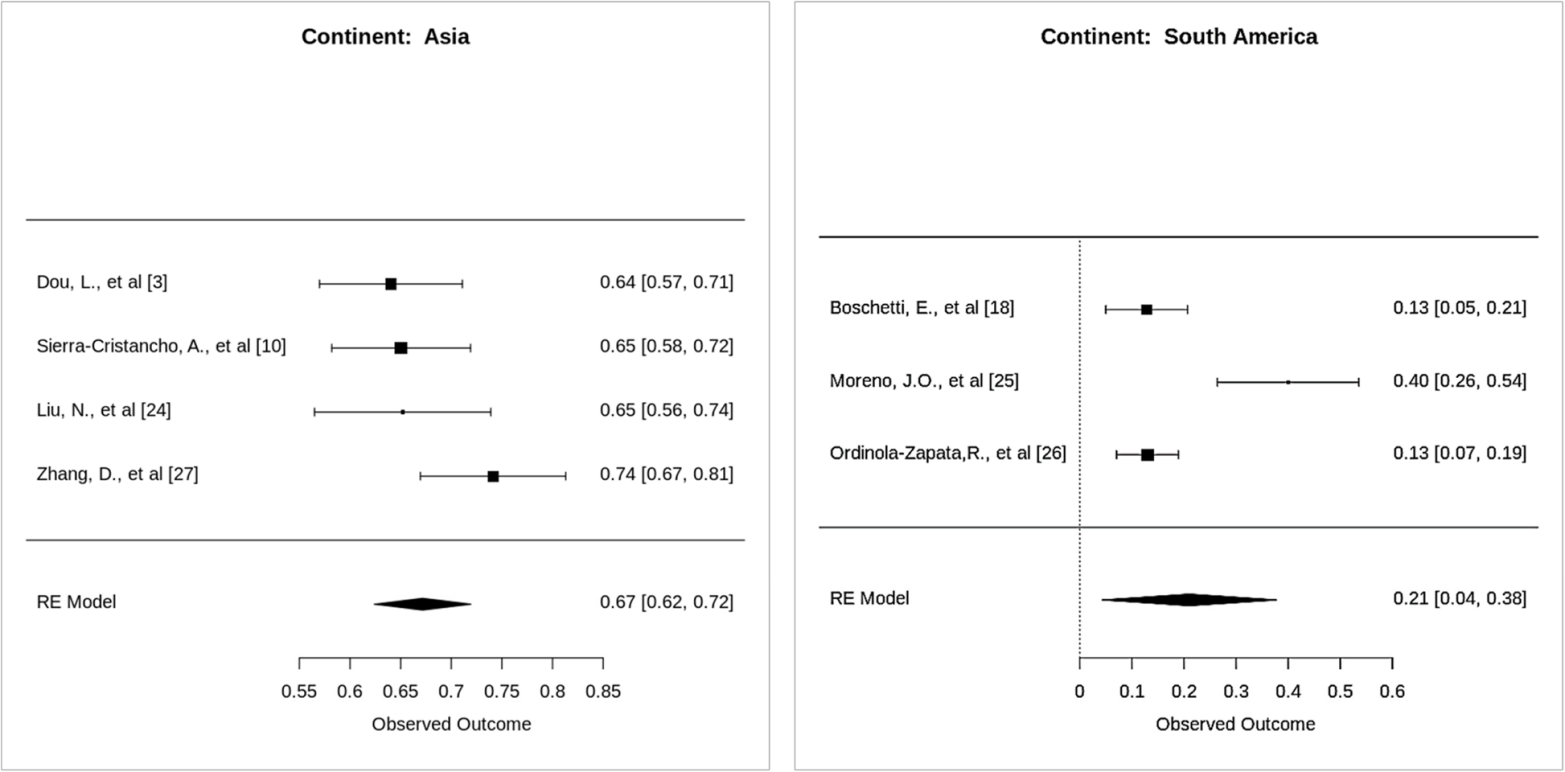
Forest plot of prevalence differences between Vertucci Type I vs Type III and Type V with different continent

The study found that the prevalence of Vertucci canal configuration was significantly higher in Asia than in other continents (*p*-value < 0.0001). The study was conducted in different countries in Asia, and the occurrence difference of Vertucci type I root canal configuration ranged between 1.12% and 6.3%. The plot shows a wide range of differences in prevalence between studies, from -20.36% to 73.43%. This suggests much variation in the prevalence of Vertucci type I, type III, and type V root canal configuration across different studies. The plot also shows no clear trend in the difference in prevalence. Vertucci type I canal configuration was more prevalent than type III and type V (Fig. 11).

**Fig. 12:**
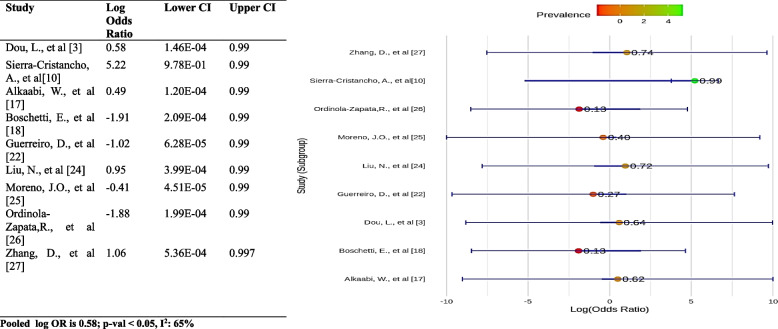
Differences of prevalence of one canal compared to more than one canal

The forest plot shows a notable difference between the incidence of one canal and more than one canal in premolars (*p* < 0.05). The pooled log OR is 0.58, meaning that the prevalence of more than one canal is 58% lower than that of one canal. The I^2^ value is 65%, indicating moderate heterogeneity between the studies. This means that some of the variation in the results is due to factors other than chance, such as differences in study design or patient populations. The results of the forest plot suggest that the prevalence of more than one canal in premolars is lower than that of one canal (Fig. 12).

**Fig. 13:**
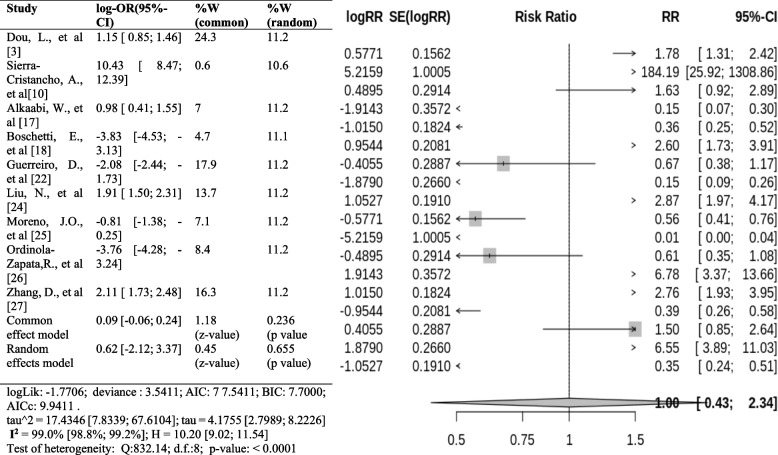
Forest plot of prevalence of one canal with more than one canal [3, 10, 17, 18, 22, 24–27]

The forest plot shows a significant difference between the occurrence of one canal and more than one canal in premolars (*p* < 0.000). The pooled log OR is 0.0913, meaning that the prevalence of more than one canal is 9.13% lower than that of one canal. The I^2^ value is 99.0%, which indicates a high amount of heterogeneity between the studies. This means that most of the variation in the results is due to factors other than chance, such as differences in study design or patient populations. The results of the forest plot suggest that the prevalence of more than one canal in premolars is lower than that of one canal (Fig. 13).

#### Prevalence of C shape canal

**Fig. 14:**
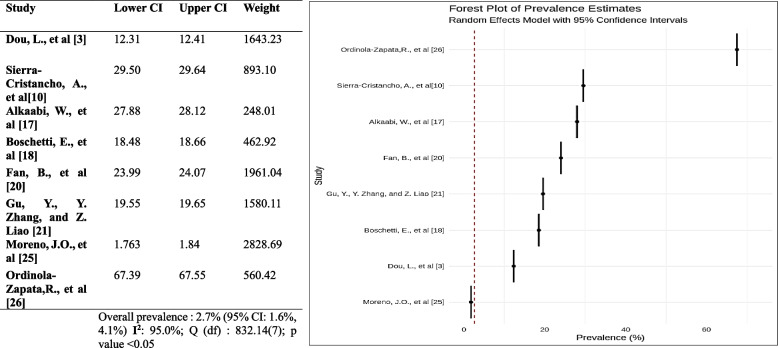
Forest plot of prevalence of c-shaped canal [3, 10, 17, 18, 20, 21, 25, 26]

The forest plot shows the estimated frequency of C-shaped canals in each study and the 95% confidence intervals (CIs) for each estimate. The pooled occurrence of C-shaped canals is 2.7% (95% CI: 1.6%, 4.1%). This means that, based on the results of this meta-analysis, approximately 2.7% of premolars have C-shaped canals. The forest plot also shows the results of a test of heterogeneity. The heterogeneity test was not significant, meaning there is no significant variation in the prevalence estimates across the studies. This suggests that the true incidence of C-shaped canals is likely to be similar in premolars across all studies. Overall, the outcome of the following meta-analysis suggested that the prevalence of C-shaped canals in premolars is relatively low (Fig. 14).

### Accessory canals

**Fig. 15:**
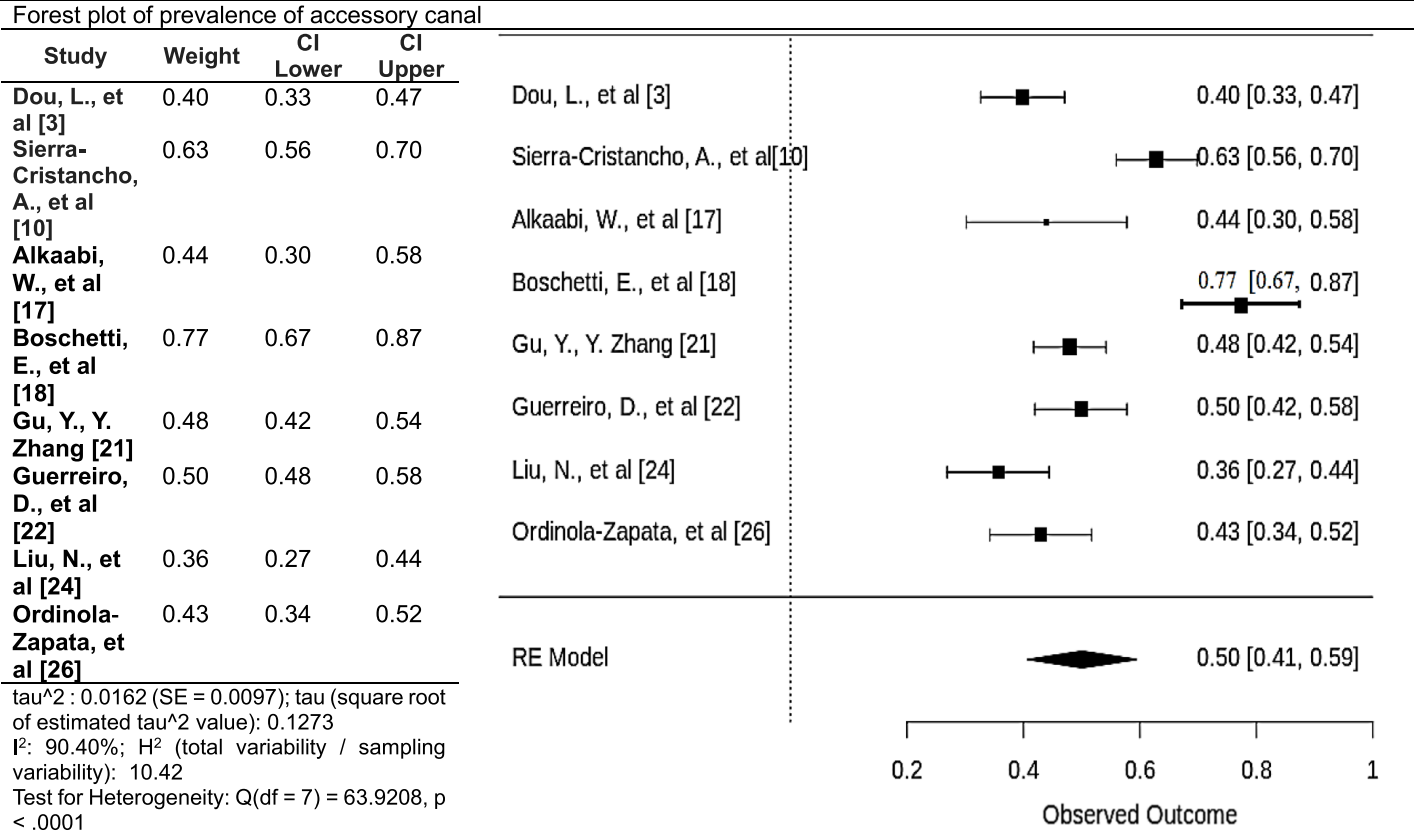
Forest plot of prevalence of accessory canals [3, 10, 18, 20–22, 24, 34]

The total pooled estimate of the prevalence of accessory canals in the premolar is 0.50(95% CI 0.41 to 0.59). This indicates that we are 95% confident that the prevalence of accessory canals in the premolars is between 41 and 59%. The high I^2^ statistic and the low p-value suggest significant heterogeneity in the estimates of the prevalence of accessory canals between the studies. The meta-analysis suggests a higher chance of accessory canals in the premolars (Fig. 15).

### Apical foramen

**Fig. 16:**
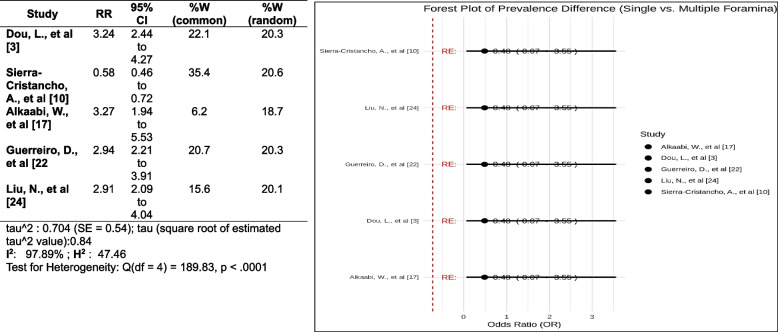
Forest plot of prevalence of apical foramen [3, 10, 17, 22, 24]

The overall pooled RR is 2.90 (95% CI 2.09 to 4.04). This means there are 2.9 times higher odds of having a single apical foramen in the premolar than multiple apical foramina. The confidence interval for the pooled RR is vast, which means there is some uncertainty about the actual value of the RR. This is because the studies involved in the present meta-analysis differed in their methods and populations. A high I^2^ statistic indicates much variation in the estimates of the RR between the studies. The I^2^ statistic in this forest plot is 75%, indicating a high degree of heterogeneity (Fig. 16).

## Discussion

Successful endodontic treatment necessitates a comprehensive grasp of both root and canal systems. Dentists must thoroughly understand the root canal structures, encompassing standard and intricate configurations, to perform complete chemico-mechanical instrumentation and subsequent root canal space filling. This knowledge is paramount for achieving optimal outcomes in endodontic procedures.

Each clinician strives to attain optimal treatment results in cases that have undergone endodontic procedures [36]. To achieve successful endodontic treatment, practitioners must possess a comprehensive understanding of both root and canal systems [37], as most root canal treatment in endodontics fails due to limited familiarity with the varied root anatomy and canal morphology [38]. This knowledge is fundamental for accomplishing complete chemico-mechanical instrumentation and proficient filling of root canal space, thereby mitigating treatment shortcomings and ensuring favourable outcomes [39].

Different methods for examining root canal structures encompass tooth clearing, tooth sectioning, conventional radiography, CBCT (Cone Beam Computed Tomography), and Micro-CT (micro-computed tomography) [35]. Microcomputed tomography, an ex vivo research method, is non-destructive and highly reproducible. It is widely recognized as the preferred approach for accurately assessing root canal morphology [40]. The present systematic review focused on comprehensively examining mandibular first premolars' root and canal morphologies through microcomputed tomography (Micro-CT). This discussion section will delve into the key findings of the review and their clinical implications, limitations of the study, and potential future directions for research in this field.

Indeed, let's delve more extensively into the discussion of the findings of this systematic review, making detailed comparisons with existing studies and highlighting the clinical implications and research gaps.

The results of this systematic review corroborate previous studies on root and canal morphologies of first mandibular premolars using Micro-CT analysis. The observed prevalence of radicular grooves (11.4%) aligns with the outcome of Thanaruengrong et al. (2019), who noted a similar incidence of 14.2% in the Thai population [41]. There is a higher prevalence of 21.42% in the Brazilian population [27], 46% in the Israeli population and 49.9% in the American population [27].

Regarding the occurrence of various Vertucci canal configurations, an increased incidence of Vertucci type I canal configuration (74.0%) in our study following the previous studies, which report 78.75% in Malaysian population [42], 78% of a Spanish population had more than one canal in mandibular first premolars [43], prevalence in Indian population was 76% [44], in Egyptian population 61.2% [45], 76.2% in Chinese population [29], 40% in Colombian population [30], and 58.2% in Jordanian population [46]. These results suggested this configuration is a predominant anatomical feature in mandibular first premolars.

Our analysis revealed a relatively low prevalence of C-shaped canals in mandibular first premolars, estimated at 2.7%. This finding aligns with the observations in the Iranian population, where rates of 1.4% [12] and 2.4% [11] were reported. However, significant variations in C-shaped canals were noticed when compared to studies conducted in different populations. For instance, studies in the USA reported a prevalence of 14% [47], while studies in India, prevalence rates of 0.92% [48] and 10% were documented [13], and the Finnish population reported a prevalence of 9% [33] and Chinese population studies, which reported a high prevalence rates of 24% [21] and 27.8% [34]. Astonishing to the above observation, the studies of Saudi [8] and Iranian populations [49] had no C-shaped canals in mandibular first premolars.

These disparities in the incidence of C-shaped canals across different populations may be attributed to various factors, including different races, sample size variations, analysis techniques, and variations in the application of statistical parameters. The intricate nature of root canal morphology and the influence of genetic and environmental factors can contribute to these observed discrepancies.

Our systematic review corroborates previous studies' findings on accessory canals and apical foramina. The estimated prevalence of accessory canals (0.50) aligns with the observations from the literature where various studies have specified that 11.53% to 46% of first mandibular premolars have multiple root canals [50], emphasizing that these anatomical variations should be anticipated and managed during root canal procedures.

Similarly, the significantly higher odds of a single apical foramen compared to multiple apical foramina, as observed in the present study, corresponds with the outcome of Cleghorn et al., who found single apical foramen in 78.9% of the teeth in a meta-analysis of root canal morphology studies [51]. This suggests clinicians should be prepared for variations in apical foramina anatomy, as multiple foramina may require different treatment approaches.

The present systematic review and meta-analysis findings provide valuable insights into the root canal morphologies of mandibular first premolars. Clinically, these insights can guide endodontists in their approach to diagnosis, treatment planning, and execution. For instance, the higher incidence of Vertucci type I canal configuration underscores the importance of considering this as the default configuration during endodontic procedures. Additionally, radicular grooves and accessory canals should be carefully assessed and addressed during treatment to ensure optimal outcomes.

In this systematic review, we have discussed the anatomical variations of root canal morphology based on the available literature. It is essential to acknowledge that root canal anatomy can exhibit variations between individuals and different populations and races. The impact of population-specific variations on root canal morphology is a topic of significant interest in endodontics, and we believe it warrants further consideration.

While our analysis included studies from various geographic regions, including China, Brazil, Chile, the United Arab Emirates, Colombia, and Malaysia, it is essential to recognize that the anatomical characteristics of teeth may differ among these diverse populations. These differences can be attributed to genetic factors, environmental factors, and evolutionary adaptations, among others. Population-specific variations in root canal morphology have been previously reported in the literature. For instance, studies have indicated that Asian populations may exhibit variations in root canal anatomy compared to Caucasian populations. These differences may extend to variations in the number of canals, canal curvatures, and accessory canals. Similarly, African, South American, and Middle Eastern populations may also display unique anatomical features in root canal systems. These variations can affect the diagnosis and treatment planning of endodontic procedures.

However, it is essential to note that the included studies in our review did not always provide detailed population-specific data. This limitation highlights the need for future research endeavours to comprehensively explore the influence of population and race on root canal anatomy. Conducting large-scale, multicenter studies encompassing diverse populations and employing advanced imaging techniques such as micro-computed tomography (Micro-CT) could shed further light on these variations. Understanding population-specific variations in root canal morphology is essential for clinical practice and advancing the field of endodontics as a whole. Dentists and endodontists should be aware that the root canal anatomy they encounter may not always conform to traditional textbook descriptions, particularly when treating patients from diverse backgrounds. Tailoring treatment approaches based on population-specific data may improve the success rates of endodontic procedures and enhance patient care.

While this systematic review provides valuable insights, it is essential to acknowledge its limitations. The study primarily focused on in-vitro studies, as micro-CT can’t be used on patients and clinics, and the data from in vivo studies might have provided a broader perspective on root canal morphologies. Additionally, the review mainly encompassed studies published in English, potentially excluding relevant non-English publications. Moreover, the inherent variability in Micro-CT imaging parameters across studies may introduce some bias in the results. The revised version of the earlier published risk of bias assessment tool was used in the current systematic review to evaluate the quality assessment of included (13) studies [18]. It mainly included five objectives, which were assessed by giving responses like yes (adequate), unclear (not specified) and no (inadequate). The objectives were calculation of sample size, reporting the quality of data by determining the various factors like scanning machine, voxel size, software and technique used, description of results with proper features like evaluation of root canal configuration by different classification, accessory canals, shaped canals, grooves, and isthmus to validate the results, reliability of an observer to minimize the errors and improve the quality of data analysed, and attrition bias which represents the sample loss in a specific region instead of generalizing it. Based on the above response, the studies were classified as low, moderate, and high risk of bias.

In Micro-CT studies, calibration and image quality are paramount for accurate results. Calibration ensures accurate measurements, while image quality affects the visibility of anatomical details. These factors are particularly crucial in studies like ours exploring root canal anatomy. Calibration Ensures Accuracy: Calibration involves translating pixel values into physical measurements. Without it, quantifying features like root canal dimensions becomes unreliable. Proper calibration relies on reference standards to correct image distortions. Image Quality Is Key: High-quality images are essential for precise root canal analysis. Poor quality can obscure details and lead to inaccurate conclusions. Image quality depends on X-ray settings, specimen preparation, and segmentation accuracy. Implications for Our Review: In our systematic review, the credibility of findings hinges on calibration and image quality. Well-documented calibration and high-quality images enhance reliability, while deficiencies in these areas introduce uncertainty.

The findings of this systematic review underscore the importance of considering root canal morphologies when planning and performing endodontic procedures. Future research in this field should address this study's limitations by incorporating a more diverse range of studies and considering non-English publications. Furthermore, investigating additional factors such as age, gender, and ethnicity that may influence root canal anatomy can provide a more comprehensive understanding. Additionally, developing innovative imaging techniques and tools can enhance the precision and accuracy of root canal assessments, further improving the success rates of endodontic treatments.

In the case of observational studies, especially those involving anatomical evaluations, sample size calculations may not be applicable in the same way as in clinical trials or experimental studies. Observational studies often involve analysing existing data or assessing naturally occurring phenomena, making it challenging to predetermine sample sizes based on traditional statistical power considerations. However, we acknowledge that the absence of sample size calculations in the included studies represents a valid limitation of this systematic review. Sample size calculations are a crucial component of research design, primarily in experimental studies, to ensure adequate statistical power and the ability to detect meaningful effects. In our review, most of the studies did not report the use of sample size calculations, which could potentially impact the precision and generalizability of their findings.

It is essential to recognize that the absence of sample size calculations should be viewed in the context of the study design. While these calculations may not be directly applicable to observational studies, their omission does limit the ability to assess the adequacy of sample sizes and the potential for type II errors (i.e., failing to detect actual effects due to inadequate sample sizes). As a result, we highlight the need for future research in this area to consider sample size calculations where appropriate. Although observational studies may not adhere to the same principles as clinical trials, researchers should strive to optimize the robustness and reliability of their findings. This may involve consulting with statisticians or methodologists to determine whether sample size calculations are feasible or relevant based on the research question and study design.

In conducting this systematic review, we aimed to comprehensively assess the existing literature on anatomical variations in dental and endodontic structures. However, it is essential to acknowledge and discuss potential limitations that may affect the scope and inclusiveness of our findings. One noteworthy limitation of our study is the potential for language bias. We primarily focused our search on English-language publications, which might have introduced a bias favouring studies published in English-speaking regions. This choice was made for accessibility and comprehensibility, as English is widely considered the global academic language.

While we conducted a systematic and thorough search within the English-language literature, we recognize that valuable research on this topic may exist in languages other than English. By restricting our search to English-language publications, we may not have captured the full spectrum of available evidence, potentially omitting important insights from non-English-speaking regions. To address this limitation, we recommend that future systematic reviews in this area consider adopting a more inclusive approach to language selection during the literature search. Researchers should be encouraged to extend their search criteria to encompass publications in languages other than English, mainly if dental and endodontic research is known to be prevalent in specific non-English-speaking regions. This approach can help reduce the risk of language bias and ensure a more comprehensive analysis of the available literature.

Furthermore, collaboration with colleagues or experts proficient in relevant non-English languages can be instrumental in overcoming language barriers and facilitating the inclusion of studies published in different linguistic contexts.

## Conclusion

This systematic review and meta-analysis have provided valuable insights into the Micro-CT characterization of root and root canal morphology in mandibular first premolars. Through an extensive examination of existing literature, we have synthesized data from numerous studies to enhance our understanding of this critical aspect of endodontics.

Our findings reaffirm the substantial anatomical variations within this tooth type, highlighting the importance of individualized treatment strategies. The detailed quantitative data presented in this review serve as a valuable resource for clinicians and researchers alike, offering a comprehensive reference for treatment planning, instrumentation, and clinical decision-making.

Moreover, we have ensured that the conclusions remain concise and clear as initially presented. The systematic nature of our review, coupled with rigorous data analysis, enhances the robustness of these conclusions. We emphasize the need for clinicians to consider the diverse anatomical configurations that mandibular first premolars may exhibit, underlining the significance of careful preoperative assessment.

As our understanding of dental anatomy evolves, clinical practice must adapt accordingly. This systematic review contributes to the existing knowledge base and underscores the ongoing need for interdisciplinary collaboration, advanced diagnostic modalities, and patient-centric approaches in endodontics. Ultimately, the insights gathered here aim to improve the quality of patient care, ensuring that each mandibular first premolar receives personalized attention based on its unique anatomical characteristics. We anticipate that these findings will guide future research endeavours and further enhance the clinical outcomes of endodontic treatments involving mandibular first premolars.

## Data Availability

All data were collected or calculated from the published included studies.
